# [Retracted] Inhibition of TLR4 protects rat islets against lipopolysaccharide-induced dysfunction 

**DOI:** 10.3892/mmr.2025.13622

**Published:** 2025-07-16

**Authors:** Xiao Wang, Qin Min Ge, Fan Bian, Yan Dong, Chun Mei Huang

Mol Med Rep 15: 805–812, 2017; DOI: 10.3892/mmr.2016.6097

Following the publication of the above paper, it was drawn to the Editor's attention by a concerned reader that, regarding the lower two flow cytometric plots shown in Fig. 3A on p. 809 (namely, the plots for the ‘anti-TLR4+LPS’ and ‘TLR4-shRNA+LPS’ experiments), these appeared to show similar groupings of dots, which would not have been anticipated if these experiments had been performed discretely under different experimental conditions, suggesting a fundamental flaw either in the way in which these experiments were performed, or in how the results were outputted.

The Editor of *Molecular Medicine Reports* has decided that this paper should be retracted from the Journal on account of a lack of confidence in the presented data. The authors were asked for an explanation to account for these concerns, but the Editorial Office did not receive a reply. The Editor apologizes to the readership for any inconvenience caused.

